# CD137 Is Induced by the CD40 Signal on Chronic Lymphocytic Leukemia B Cells and Transduces the Survival Signal *via* NF-κB Activation

**DOI:** 10.1371/journal.pone.0064425

**Published:** 2013-05-16

**Authors:** Yukana Nakaima, Ken Watanabe, Takatoshi Koyama, Osamu Miura, Tetsuya Fukuda

**Affiliations:** 1 Department of Hematology, Graduate School of Medical and Dental Sciences, Tokyo Medical and Dental University, Tokyo, Japan; 2 Laboratory Molecular Genetics of Hematology, Graduate School of Health Sciences, Tokyo Medical and Dental University, Tokyo, Japan; Istituto Superiore di Sanità, Italy

## Abstract

CD137 is a member of the tumor necrosis factor receptor family that is expressed on activated T cells. This molecule provides a co-stimulatory signal that enhances the survival, and differentiation of cells, and has a crucial role in the development of CD8 cytotoxic T cells and anti-tumor immunity. Here we report that CD137 expression is also induced on normal or malignant human B cells by CD40 ligation by its ligand CD154. This CD137 induction was more prominent in chronic lymphocytic leukemia (CLL) cells than in other types of B cells. CD137 stimulation on B cells by its ligand induced the nuclear translocation of p52 (a non-canonical NF-κB factor). In agreement with this finding, expression of the survival factor BCL-XL was upregulated. Consequently, the CD137 signal augmented the survival of CD154-stimulated CLL B cells *in vitro*. This unexpected induction of CD137 on B cells by CD40 signal may influence the clinical course of CLL.

## Introduction

The microenvironment in lymphatic organs plays an important role in humoral and cellular immunity. In addition to the effects elicited by soluble factors such as cytokines and chemokines, various receptors are activated by the ligands present on “bystander cells,” and transduce the signals that are crucial for the proliferation, survival and differentiation of cells. These co-stimulatory receptors are expressed constitutively or induced on lymphocytes and contribute to the appropriate immunological response.

CD137 is a co-stimulator that belongs to the tumor necrosis factor receptor superfamily. CD137 is induced on activated T cells by the TCR signal, and this augments the proliferation and differentiation of these T cells. CD137 expression is induced by antigenic activation on CD4 and CD8 T cells, and plays a key role in the development of CD8 cytotoxic T cells [1]. It has been reported that stimulation via this receptor results in not only blockade but also reversal of anergy status [2]. In accordance with this hypothesis, an increasing number of reports have suggested the efficacy of agonistic reagents of CD137 to induce tumor immunity for cancer therapy in animal models. Owing to these promising results, several clinical studies using agonistic anti-CD137 antibodies for solid malignancies are ongoing [3]. CD137 is also expressed on dendritic cells, NK cells, and hematopoietic progenitor cells [4]–[9], suggesting the possibility of multi-layered effects of this type of targeted immunotherapy [10]. A recent report of CD137 expression on atherosclerotic endothelial cells also suggests the possibility that its expression is induced in unrecognized cell types [11]. Therefore, examining the possible expression of this receptor and its ligand (CD137L) on tumor cells is important.

The microenvironment also influences lymphoid tumor development. Recent studies had revealed the importance of T cells, particularly follicular helper T cells, macrophages, and follicular dendritic cells, for the development of follicular lymphoma (FL) [12]. The microenvironment also influences the prognosis of FL; microarray data revealed that more T cells rather than macrophages in tumor tissues result in better clinical courses [13].

Chronic lymphocytic leukemia (CLL) is also influenced by its microenvironment. It has been reported that CLL cells proliferate mainly in pseudofollicular proliferation centers in lymphatic organs *via* interactions with activated T cells [14]. Although CD154–CD40 interaction may play a pivotal role in this microenvironment, other mechanisms that support CLL proliferation are not completely elucidated.

Here we report that CD137 is induced on normal and malignant B cells by the CD40 signal. CD137 is prominently induced on CLL B cells, and may play a role in the prevention of apoptosis in these cells through activation of NF-κB.

## Methods

### Ethics Statement

After written informed consent was obtained in compliance with the Declaration of Helsinki, samples (peripheral blood, bone marrow, lymph node, ascites, and pleural effusions) were collected from patients. Approval was obtained from the ethical committee of Tokyo Medical and Dental University.

### Cell preparation

Leukemias and lymphomas were classified according to the WHO classification. CLL was diagnosed according to the National Cancer Institute Working Group guidelines [15]. Peripheral blood mononuclear cells (PBMCs) from patients or healthy voluntary adult donors were isolated through density-gradient centrifugation from freshly collected blood samples. Tumor cells from the bone marrows, lymph nodes, and pleural effusion were obtained by diagnostic procedures. Cells were resuspended in Cellbanker (Mitsubishi Chemical Medicine) for viable frozen storage and used after thawing for experiments. All tumor cells were CD19 positive, and the percentages of tumor cells were more than 95% of CD19 positive cells. Activated T cells were prepared using anti-CD3/CD28 beads (DYNAL) as described previously [16]. HeLa, BJAB, Ramos, Jurkat, CHO, and CD32 L cells were obtained from the American Type Culture Collection. Human CD137 and CD137L cDNAs were cloned by RT-PCR, subcloned into pcDNA3 or pcDNA4HisMax plasmids, and transfected into BJAB, Ramos, Jurkat, and CHO cells. HeLa CD154 transfectant (HeLa-CD154) was generated as described previously [17].

### Antibodies and reagents

Allophycocyanin (APC)-labeled anti-CD19 antibody, FITC-labeled anti-CD3 or anti-CD95 antibody, PE-labeled murine anti-human CD137 or CD137L antibody, biotin anti-CD54 with FITC-labeled streptavidin and relevant isotype control IgG1 (BD PharMingen) were used for flow cytometry. We used murine anti-p52 and rabbit anti-p65 antibodies (Upstate Biotechnology), rabbit anti-BCL-XL (Becton Dickinson), murine anti-YY1, and murine anti-α-tubulin (Santa Cruz). Horseradish peroxidase-conjugated anti-murine or anti-rabbit antibody (GE Healthcare) was used as the secondary antibody. For EMSA, we used the aforementioned anti-p52 antibody, and anti-p50 and anti-p65 antibodies (Santa Cruz). LPS, PMA, ionomycin, and SP600125 were obtained from Sigma. Oligodeoxynucleotides (ODNs) were synthesized by Integrated DNA Technologies. Anti-human IgM antibody (Southern Biotech) as well as anti-CD40 and anti-CD154 antibodies (BD PharMingen) were used for cell culture. The matrix metalloprotease inhibitor GM6001 was from Millipore.

### Flow cytometry

FACS data obtained by FACSCalibur (Becton Dickinson) were analyzed with FlowJo software (Tree Star). For the survival assay, the viability was determined by mitochondrial transmembrane potential using 3,3′-dihexyloxacarbocyanine (DiOC6; Invitrogen) as described previously [18]. CLL B cells were stained with PKH-26 (Sigma) according to the manufacturer's instructions. Labeled cells were co-cultured with HeLa-CD154 for 24 h and thereafter with CHO control or CD137L transfectant. On days 2, 4, 6, and 8, cell viability was determined.

### RT-PCR

PBMCs from healthy donors or CLL patients were used. The percentage of CLL-B cells analyzed was higher than 95% by flow cytometry. RNA was isolated using Trizol (Gibco BRL). Reverse transcription using an oligo dT primer was carried out with a SuperScript II kit (Invitrogen). The synthesized cDNA was amplified with primers specific for human CD137 (forward: 5′-GTGCCAGATTTCATCATGGG-3′; reverse: 5′-CAACAGCCCTATTGACTTCC-3′). Expression of β-actin was used for quantitative normalization.

### Immunoblot analysis

Cellular proteins were divided into cytoplasmic and nuclear fractions using hypotonic buffer as described previously [19]. Proteins were quantified using a DC protein assay kit (BioRad). Equivalent quantities of proteins were subjected to SDS-PAGE and transferred onto Immobilon-P membranes (Millipore).

### EMSA

EMSA analyses were conducted according to the manufacturer's protocols of the DIG Gel Shift Kit, 2nd Generation (Roche). Briefly, nuclear proteins were prepared with nuclear extraction buffer (20 mM HEPES, 500 mM NaCl, 1.5 mM MgCl_2_, 0.5 mM EDTA, 0.5 mM DTT, 25% glycerol) and 2 µg of proteins were applied for each reaction. The labeled probe employed was annealed using oligonucleotides corresponding to the NF-κB (5′-AGTTGAGGGGACTTTCCCAGGC-3′) or NF-Y (5′-ATTTTTCTGATTGGTTAAAAGT-3′) binding site.

### Immunofluorescence

Cells were deposited on MAS-coated glass slides by cytospin centrifugation and then fixed in 4% paraformaldehyde. Cells were stained with the appropriate primary antibody and donkey anti-murine antibody conjugated with Cy5 (Jackson ImmunoResearch). The slides were mounted using ProLong Gold antifade reagent with DAPI (Molecular Probes). Images were obtained using a BZ-8000 fluorescence microscope (Keyence).

### Statistical Analysis

The significance of the differences between groups was determined by Mann–Whitney U test, Student's t test or Student's paired *t*-test using the GraphPad Prism Software. P-values <0.05 were considered statistically significant.

## Results

### Induction of CD137 expression on human B cells by activated T cells

**Figure 1:**
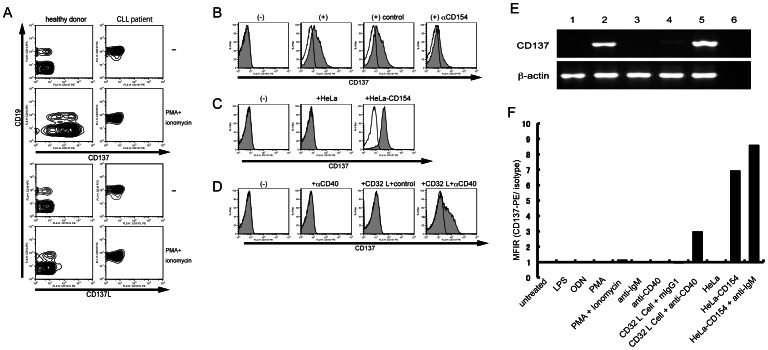
CD40 signal induces CD137 expression on B cells. (A) PBMCs from a healthy donor or a CLL patient were stimulated with or without 10 ng/ml PMA and 1 mM ionomycin for 48 h, as indicated. Cells were then incubated with anti-CD137-PE or anti-CD137L-PE monoclonal antibodies and APC-labeled anti-CD19 antibody for flow cytometric analysis. (B) CLL B cells were cultured alone (−) or co-cultured with T cells activated by magnetic beads coated with anti-CD3/CD28 antibodies (+). The blocking monoclonal antibody against CD154 or control monoclonal antibody was added in the culture, as indicated. Expression of CD137 on CD19-positive CLL cells was analyzed by flow cytometry. (C) CLL B cells were cultured alone (–) or co-cultured with HeLa control or HeLa-CD154, as indicated, for 24 h before analysis of CD137 expression. (D) CLL B cells were analyzed after being co-cultured for 24 h with or without CD32 L cells that had captured agonistic anti-CD40 or control isotype murine IgG antibody, as indicated. (E) RT-PCR was done using various samples as follows: lane 1, CLL B cells co-cultured with HeLa control cells; lane 2, CLL B cells co-cultured with HeLa-CD154; lane 3, HeLa-CD154 cells alone; lane 4, PBMCs from a healthy donor alone; lane 5, PBMCs from a healthy donor stimulated with PMA and ionomycin; lane 6, negative control without the RT reaction. After culture for 48 h, RNA was extracted by Trizol for RT-PCR analysis. RT-PCR products obtained with primers for CD137 or β-actin (as the quantitative control), are shown as indicated. (F) CLL B cells were cultured with the indicated stimulator(s). After 24 h, cells were subjected to FACS analysis. MFIR is plotted, which is calculated by dividing the mean fluorescence intensity of cells stained with a PE-CD137 monoclonal antibody by that of cells stained with a PE conjugated isotype control monoclonal antibody.

We first examined the expressions of CD137 and its ligand on PBMCs from a healthy donor after PMA/ionomycin treatment because it is known that CD137 is induced on human T cells after such stimulation. CD137 induction could be detected on CD19-positive B cells as well as on CD19-negative cells (Fig. 1A). However, CD137 induction was not observed when PBMCs from a CLL patient were used (Fig. 1A). We speculated that activated T cells induced CD137 on B cells because few T cells were included in CLL PBMCs. Therefore, the CLL B cells were co-cultured with CD3/CD28-stimulated allo-T cells. The activated T cells clearly induced CD137 expression on CLL B cells (Fig. 1B). Functional CD137L expression on CLL B cells has been reported [20], but such expression was not detected in our CLL cases even after PMA/Ionomycin treatment, possibly due to low sensitivity.

### Induction of CD137 on CLL B cells by the CD40 signal

CD40–CD154 is a key element of the interaction between activated T and B cells. To examine if this interaction is involved in CD137 induction on B cells, we added the blocking antibody for CD154 in the mixed culture of CLL B cells and activated T cells. This antibody significantly reduced the CD137 induction on B cells, whereas an isotype control antibody did not show any significant effect (Fig. 1B). We further confirmed the involvement of the CD40–CD154 interaction in CD137 induction using CD154-transfected HeLa cells (HeLa-CD154). Co-culture with HeLa-CD154 cells, but not with parental HeLa cells, could strongly induce CD137 expression on CLL B cells (Fig. 1C). Furthermore, the agonistic anti-CD40 antibody crosslinked with CD32-expressing murine fibroblast cells (CD32 L cells) also induced CD137 on CLL B cells, whereas the antibody alone could not induce CD137 expression (Fig. 1D).

The intrinsic induction of CD137 expression in B cells by the CD40 signal was demonstrated by RT-PCR analysis, which revealed that *CD137* was induced at the mRNA level in CLL B cells (Fig. 1E). The sequencing analysis further revealed that the soluble form of *CD137* (*sCD137*), generated by alternative splicing [21], [22], was also induced in addition to the membranous type (NM_001561) in CLL B cells (data not shown).

Next, we checked the inducibility of CD137 by other stimuli for B cells, including LPS, ODNs, PMA, ionomycin, and anti-IgM antibody. CD40 stimulation was the only factor that induced CD137 expression on CLL B cells (Fig. 1F). The addition of anti-IgM could slightly augment CD137 induction by CD154, although it could not induce CD137 by itself.

### Prominent induction of CD137 on CLL B cells

**Figure 2:**
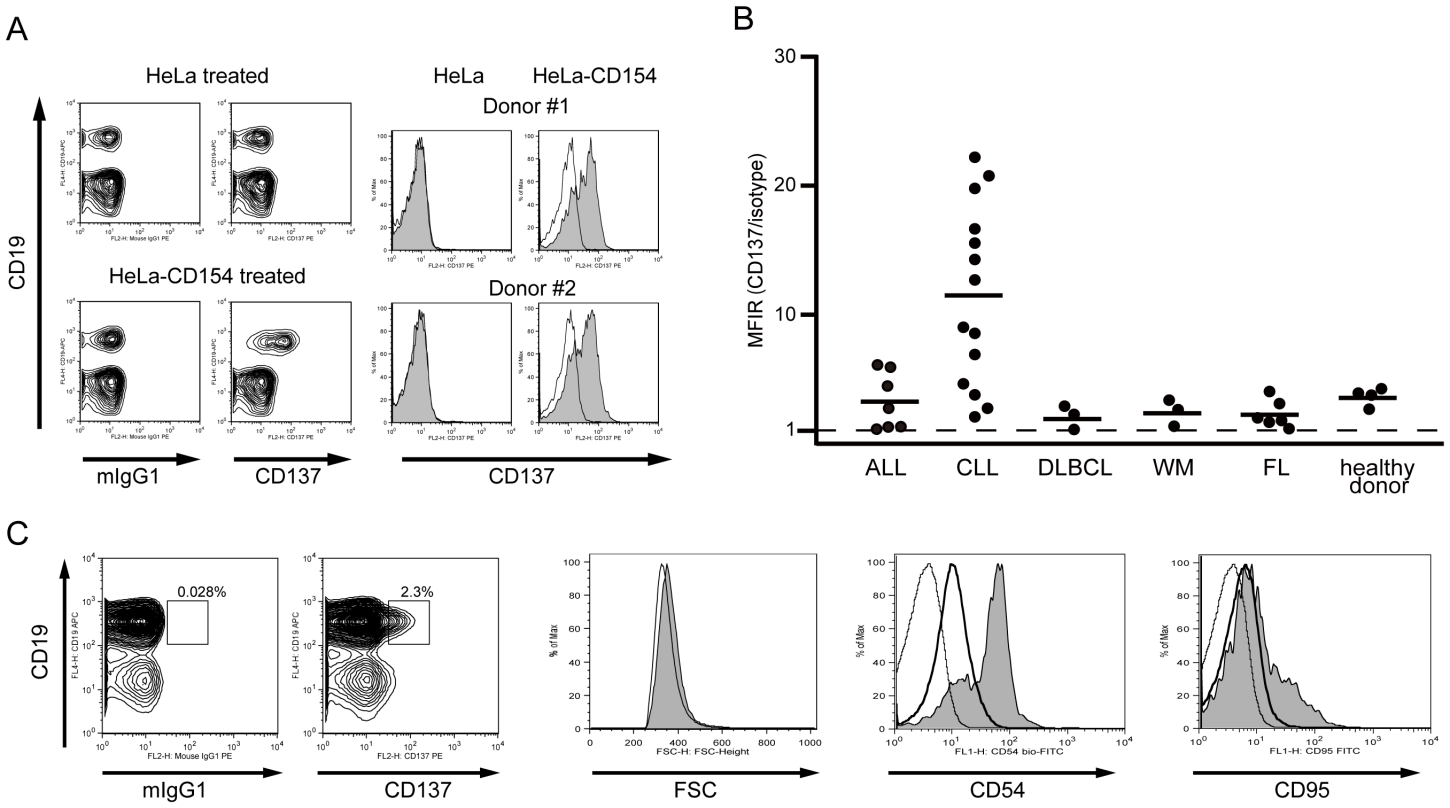
CD137 induction on B cells derived from patients with various B-cell malignancies. (A) PBMCs from a healthy donor were co-cultured with parental HeLa cells or cells expressing CD154 for 24 h, as indicated, and subjected to flow cytometric analysis for expression of CD19 and CD137. Histograms for CD137 expression on CD19-positive cells from 2 healthy donors are shown on the right side (shaded histogram). The murine IgG1 control is represented by the open histogram. (B) Expression of CD137 on B cells derived from patients with B-cell malignancies as well as that from healthy donors was analyzed after co-culture with HeLa-CD154. Each symbol represents an individual patient. The horizontal bars indicate the mean values of each group (ALL, 3.3; CLL, 11.4; DLBCL, 2.1; WM, 2.4; FL, 2.3; healthy donor, 3.7). Statistical analysis performed using the Mann–Whitney U test revealed that CD137 expression in the CLL group was significantly higher than that in non-CLL cases (p<0.001) or healthy donors (p = 0.001). (C) Expression of CD137 on PBMCs was analyzed in a patient with CLL without stimulation. Histograms for FSC as well as for expressions of CD54 and CD95 are shown on the right. Shaded, open, and dotted histograms represent CD137-positive, CD137-negative B cells, and isotype control, respectively.

To evaluate whether CD137 induction by the CD40 signal is unique to CLL B cells, PBMCs from healthy donors were stimulated with HeLa-CD154 cells. CD137 induction was clearly observed also on normal peripheral B cells (n = 4, Fig. 2A and 2B). Next, we examined CD137 induction on various types of malignant B cells derived from patients with B-cell acute lymphoblastic leukemia (ALL; n = 7), CLL (n = 14), diffuse large B-cell lymphoma (DLBCL; n = 3), Waldenström macroglobulinemia (WM; n = 3), and FL (n = 6). Each of the primary cells was co-cultured with HeLa-CD154 and analyzed by FACS. The induction of CD137 was clear (Mean Fluorescence Intensity Ratio (MFIR) >1.5) in 27 cases but not in 6 cases (Fig. 2B, Table S1 and S2). Notably, CD137 induction was observed clearly in all CLL cases, and its average value was significantly higher than that of non-CLL cases (p<0.001) or healthy donors (p = 0.001).

This prominent induction of CD137 on CLL B cells prompted us to examine the *in vivo* induction of CD137 in CLL patients. We analyzed CD137 expression on 10^6^ CLL B cells from the peripheral blood of 7 patients by flow cytometry. In 2 patients, CD137-positive cells could be detected as 2.3% and 0.76% of the CD19-positive and CD3-negative population, respectively (Fig. 2C and data not shown). In the other 5 samples, the percentage of CD137-positive cells was <0.5% and they were indistinguishable from those counted in the non-specific reaction. CD137-positive cells were slightly larger than CD137-negative CLL cells as judged by FSC intensity, and expressed CD54 and CD95 at higher levels (Fig. 2C), suggesting that they might have recently been activated.

### Non-canonical NF-κB activation by CD137 in B cells

**Figure 3:**
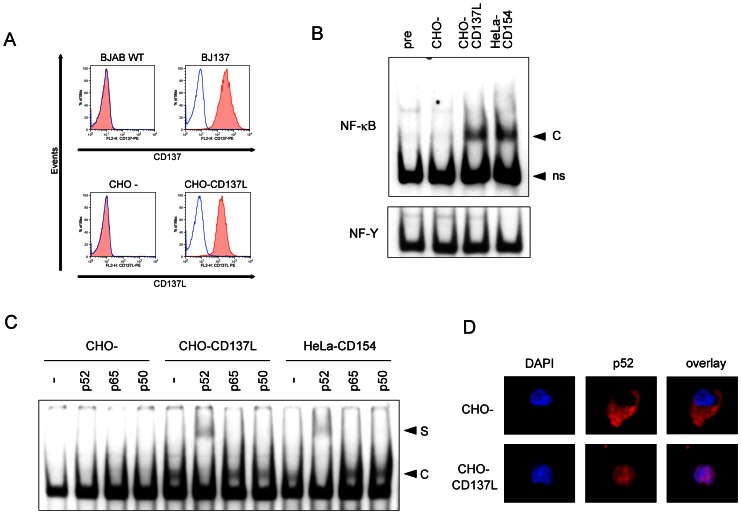
NF-κB activation in BJ137 cells. (A) Parental BJAB (BJAB WT) and CHO (CHO-) as well as their transfectants expressing CD137 (BJ137) and CD137L (CHO-CD137L) were subjected to flow cytometric analyses for CD137 and CD137L, as indicated. BJ137 was not cultured (pre) or co-cultured with indicated cells for 24 h and then subjected to the following analyses. (B) NF-κB binding activity in BJ137 was examined by EMSA with a DIG-labeled probe of the NF-κB site or a control probe (NF-Y). (C) Nuclear extracts were pre-incubated with antibodies against indicated proteins before incubation with the NF-κB probe. C: DNA–protein complex. S: supershifted band. ns: non-specific band. (D) The nuclear translocation of p52 was visualized by immunofluorescence using fluorescence microscopy. Cells were labeled with DAPI (blue) to visualize the nuclei and stained with anti-p52 conjugated with Cy5 (red).

**Figure 4:**
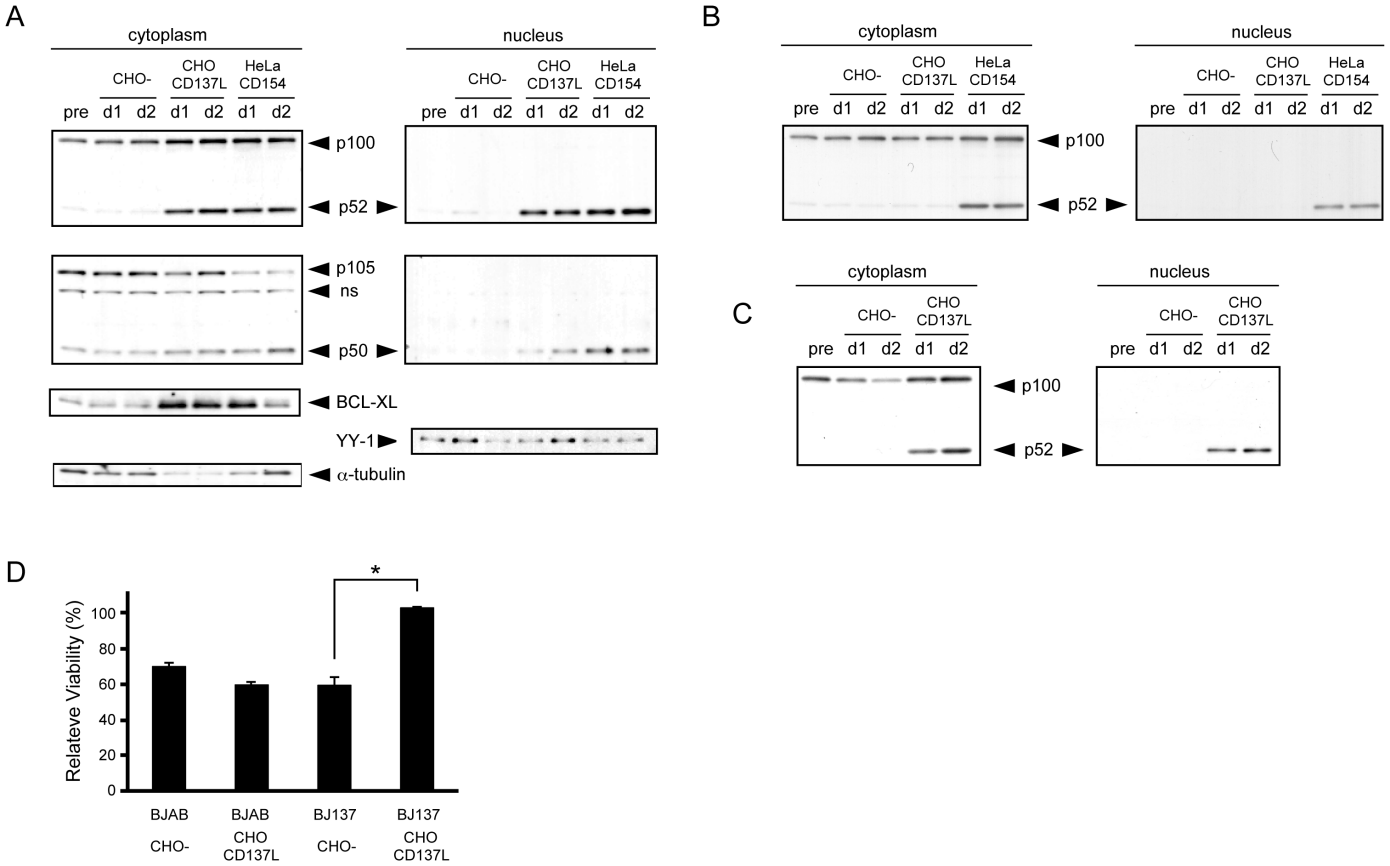
The CD137–CD137L interaction leads to the nuclear translocation of NF-κB2 and p52. (A) Proteins were extracted from BJ137 on day 1 (d1) and day 2 (d2) as cytoplasmic and nuclear fractions, and evaluated by immunoblot analysis using antibodies against the indicated proteins. (B) BJAB cells without CD137 expression (BJAB WT) and the (C) Jurkat T cell line transfected with CD137 (Jurkat-CD137) were co-cultured with the indicated cell lines for 1 (d1) or 2 (d2) days. Cytoplasmic and nuclear extracts were subjected to immunoblot analysis with anti-p52 antibody. The positions of proteins are indicated. Non-specific bands are indicated as ns. (D) Parental BJAB and BJ137 cells were cultured on CHO or CHO-CD137L cells over night. Cells were subsequently cultured for 2 days with or without 30 µM of SP600125 and stained with DiOC6 for analyzing the viability. The relative viability of cells cultured with SP600125 as compared with corresponding cells cultured without the inhibitor is plotted (n = 4, *p<0.001; Student's *t*-test).

Because CD40 signal itself induces the strong activation signals in B cells, we generated a BJAB-CD137 transfectant (BJ137), as well as CHO-CD137L as a ligand presenter, to elucidate molecular events induced by CD137 signal itself and first confirmed their expressions (Fig. 3A). NF-κB activation *via* CD137 has been well documented in activated T cells [23], so we first examined NF-κB activation by EMSA. Co-culture with CHO-CD137L, as well as CD40 stimulation with HeLa-CD154, clearly induced activation of NF-κB in BJ137 cells (Fig. 3B). A supershift assay using specific antibodies revealed that NF-κB2 p52 was the major component of this activity (Fig. 3C). p52 activation was confirmed by immunofluorescence staining, which showed nuclear translocation of p52 after CD137 stimulation (Fig. 3D). In addition, immunoblot analyses after subcellular fractionation clearly revealed that the CD137 signal induced the cleavage of NF-κB2 p100 to p52 and the nuclear translocation of p52 in BJ137 cells (Fig. 4A) but not in parental BJAB cells (Fig. 4B). The canonical NF-κB member p50 derived from p105 was also translocated to the nuclear fraction, although EMSA could not detect it presumably because of the low sensitivity of this assay. As compared with CD40 stimulation, CD137 stimulation induced the nuclear translocation of p52 more predominantly than that of p50. This non-canonical NF-κB activation was not unique to this cell line because similar activation could be detected in the CD137 transfectant of another B cell line, Ramos (data not shown), as well as in a T cell line, Jurkat (Fig. 4C).

It has been reported that CD137 induces the survival factor BCL-XL through NF-κB activation [23], [24]. In accordance with these reports, BCL-XL was upregulated in BJ137 after CD137 ligation (Fig. 4A). The induction of BCL-XL was also detected after 1 day of CD40 ligation, but it was diminished on day 2. In contrast, BCL-XL was maintained on day 2 of CD137 ligation. Furthermore, CD137 ligation protected BJ137 cells, but not parental BJAB cells, from the decline in viability induced by the JNK inhibitor SP600125 (Fig. 4D), in accordance with a previous report that SP600125 induced apoptosis in various lymphoma cells, including BJAB cells, which was inhibited by BCL-XL [25].

### Protection of CLL B cells against spontaneous apoptosis *in vitro* by CD137 signaling

**Figure 5:**
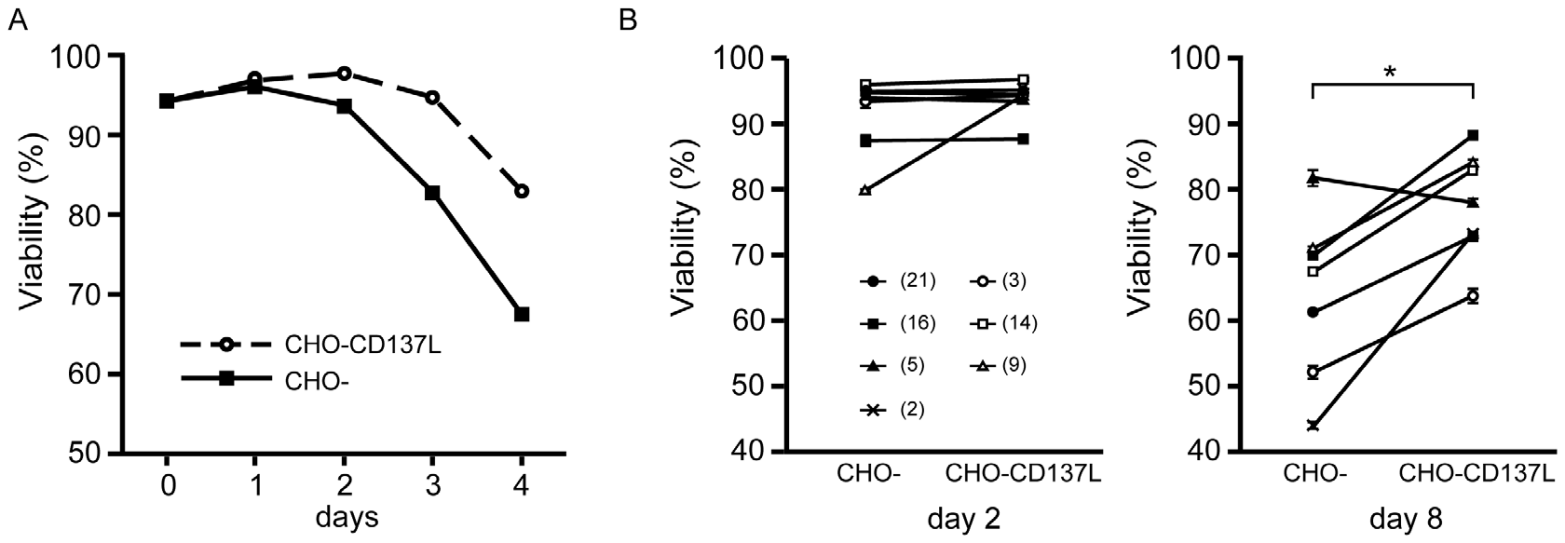
The CD137–CD137L interaction protects CLL B cells from spontaneous apoptosis. (A) PBMCs from a CLL patient were stained with PKH26. Stained cells were co-cultured with HeLa-CD154 to induce CD137 followed by co-culture with parental CHO (CHO-) or CD137L transfectant (CHO-CD137L) for the days indicated. Cells were then stained with DiOC6 and analyzed for the viability of PKH26-positive cells by flow cytometry. (B) Samples from 7 CLL patients were treated in the aforementioned manner and analyzed on day 2 and day 8. The MFIR of CD137 expression, represented in Fig. 2B, is indicated in parentheses for each sample. The viabilities on day 8 of CLL B cells cultured with CHO-CD137 were significantly higher than those cultured with CHO (*p = 0.01; Student's paired *t*-test), whereas there was no significant difference on day 2 (p = 0.3).

To address the possibility that the CD137–CD137L interaction influences the survival of primary CLL B cells, we evaluated the effect of CD137 ligation on the spontaneous apoptosis of CD40-ligated cells. Viable DiOC6-positive cells were detected at the initial phase of culture with or without CD137 stimulation, the decline in viability was attenuated with CD137 ligation thereafter (Fig. 5A). Although the difference was not obvious on day 2, the viability of CLL B cells on day 8 after CD40 stimulation was higher when co-cultured with CHO-CD137L than with the CHO control in 6 out of 7 samples (Fig. 5B). These findings suggested the anti-apoptotic effect of the CD137–CD137L interaction following CD40 ligation, while the increase in viability did not correlate well with that in CD137 expression after CD40 ligation shown in Fig. 2B.

## Discussion

In the present study, we reported that activated T cells induced CD137 on malignant as well as normal B cells *via* the CD40 signal. After we had started these experiments, Zhang et al. also reported that CD137 is induced and promotes proliferation and survival of human normal B cells [26], but CD137 expression on B cell malignancies were not known. In this study, we have revealed that most types of B cells showed this induction after CD40 stimulation, and the induction was observed most prominently on CLL B cells. The induction was very strong in a group of CLL samples, but some of them showed less inducibility. Scielzo et al. reported the presence of two groups in CLL cases: CD40L dependent and independent [27]. It is tempting to speculate that the variation in CD137 inducibility may be associated with such CD40L dependency of CLL. Furtner et al. [28] reported that sCD137 could be detected in the sera of patients with hematological malignancies, with the strongest association being observed between CLL and elevated levels of sCD137, a finding that was comparable with our results. Collectively, these data along with the finding of *sCD137* mRNA induction in CLL B cells by CD40 stimulation suggest that *CD137* expression should be induced in tumor cells in the body, resulting in elevated levels of sCD137 in CLL patients. Very recently, Buechele et al. reported that functional CD137L is expressed on CLL B cells constitutively and that it modulates the reactivity of NK cells [20]. According to this report, CLL B cells should obtain a signal *via* induced CD137 in contact with surrounding CLL B cells in a paracrine manner.

CD137 induces NF-κB activation in activated T cells. However, the relative contributions of the 2 NF-κB signaling pathways from this receptor have not been described. We have revealed that CD137 stimulation activates NF-κB *via* the canonical pathway and the non-canonical pathway in B cells as well as in T cells. Non-canonical NF-κB activation in B cells by CD40 and B cell activation factor (BAFF) has been well documented [29]–[31], but activation of this pathway in T cells is largely unknown. The canonical and non-canonical pathways may have different functions, therefore, the unique function of CD137 in cellular immunity may come from this non-canonical pathway.

Because of NF-κB activation, anti-apoptotic BCL-XL expression was upregulated in BJ137 cells, which were protected from apoptosis by CD137 ligation. It has been reported that CLL cells mainly proliferate in pseudofollicular proliferation centers in lymphatic organs *via* interactions with activated T cells [14]. The CD154–CD40 interaction may play a pivotal role in this microenvironment. However, CD40 ligation makes B cells fragile and susceptible to immunological attack [16]. Induced CD137 may have a protective role by maintaining survival in this situation in a spatiotemporally regulated manner after the CD40 signal. BCL-XL expression is observed more prominently in CLL B cells in lymph nodes as compared with those in peripheral blood [32], [33].

Absolute numbers of T cells are often increased in CLL patients, and T-cell dysfunctions are known to account for the autoimmunity or cellular immunodeficiency observed in this disease [34]. In addition, immunosurveilance for tumor progression in these patients is insufficient. This is despite the fact that CLL B cells express MHC class I and class II molecules that can present autologous tumor antigens, and it is speculated that anti-CLL T cells are produced spontaneously [35]. Suppressive cytokines such as TGF-β and IL-10 produced by CLL B cells may contribute to this anergic status [36], [37]. In addition, inadequate co-stimulatory signals, particularly from CD137 may induce this anergy. Activated anti-CLL T cells should be surrounded by CD40-activated CLL B cells that induce CD137 expression, so these T cells may not be stimulated adequately through CD137.

**Figure 6:**
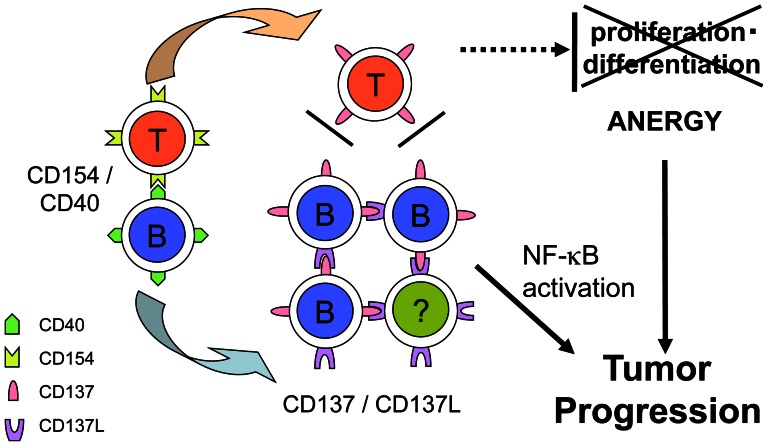
Hypothesized mechanisms for the contribution of CD137 induction to CLL progression via two pathways. First, expression of CD137 is induced on CLL B cells that are in contact with activated T cells at the pseudofollicular proliferation center. Then, the CD137–CD137L interaction induces non-canonical NF-κB activation, which may contribute to the proliferation and survival of CLL B cells in the periphery. Conversely, T cells fall into anergy because they fail to contact CD137L-expressing cells because of competition with numerous CLL B cells that express CD137.

It is tempting to speculate that CD137 induction may contribute to the development or progression of CLL in this microenvironment through two mechanisms: (i) the intrinsic induction of survival factor(s) by NF-κB activation by CD137 signaling and (ii) the inhibition of T-cell co-stimulation through CD137 by competitive binding with CD137L (Fig. 6). In a recent study, an anti-CD137 agonistic antibody demonstrated a promising anti-lymphoma effect by modulating host immunity in a murine model [38]. However, the present study raises the possibility that this therapy may also stimulate human tumor progression that is dependent upon tumor type and the timing of stimulation. These factors should be evaluated carefully in future studies.

## Supporting Information

Table S1 **Characteristics of CLL samples.**
(PDF)

Table S2 **Characteristics of non-CLL samples.**
(PDF)
