# Supplementary material for: CD137 Is Induced by the CD40 Signal on Chronic Lymphocytic Leukemia B Cells and Transduces the Survival Signal via NF-κB Activation
Source: PLoS One. 2013 May 16;8(5):e64425. doi: 10.1371/journal.pone.0064425 (PMC3655981; doi:10.1371/journal.pone.0064425)
Supplement: Table S1 — Characteristics of CLL samples. (PDF) [file pone.0064425.s001.pdf]

Table S1 Characteristics of CLL samples

Patient Number	Disease	Source	ZAP−70	IGVH mutation	CD137 induction MFIR
1	CLL	PB	−	+	2.1
2	CLL	PB	−	+	2.8
3	CLL	PB	−	+	3.8
4	CLL	PB	−	+	4.7
5	CLL	PB	−	−	6.9
6	CLL	PB	+	−	8.5
7	CLL	PB	+	−	9.0
8	CLL	PB	+	−	12.7
9	CLL	PB	−	+	14.2
10	CLL	PB	−	+	15.7
11	CLL	PB	−	+	16.6
12	CLL	PB	−	+	19.9
13	CLL	PB	−	+	20.8
14	CLL	PB	+	−	22.3

Abbreviations: CLL, Chronic lymphocytic leukemia; PB, peripheral blood
