# Supplementary material for: CD137 Is Induced by the CD40 Signal on Chronic Lymphocytic Leukemia B Cells and Transduces the Survival Signal via NF-κB Activation
Source: PLoS One. 2013 May 16;8(5):e64425. doi: 10.1371/journal.pone.0064425 (PMC3655981; doi:10.1371/journal.pone.0064425)
Supplement: Table S2 — Characteristics of non-CLL samples. (PDF) [file pone.0064425.s002.pdf]

Table S2 Characteristics of non-CLL samples

Patient Number	Disease	source	CD137 induction MFIR
15	ALL (pro B)	BM	1.1
16	ALL (pro B)	PB	1.3
17	ALL (pro B)	BM	6.1
18	ALL (common)	BM	1.3
19	ALL (common)	PB	2.7
20	ALL (common)	PB	4.4
21	ALL (common)	PB	5.9
22	DLBCL	LN	1.0
23	DLBCL	PE	2.3
24	DLBCL	LN	3.0
25	WM	LN	1.3
26	WM	BM	2.6
27	WM	BM	3.4
28	FL	LN	1.2
29	FL	PB	1.6
30	FL	LN	1.8
31	FL	LN	1.9
32	FL	PB	3.1
33	FL	PB	4.0
34	healthy donor	PB	2.8
35	healthy donor	PB	3.8
36	healthy donor	PB	4.0
37	healthy donor	PB	4.3

Abbreviations: ALL, Acute lymphoblastic leukemia; FL, Follicular lymphoma; DLBCL, Diffuse large B-cell lymphoma; WM, Waldenström macroglobulinemia; BM, bone marrow; PB, peripheral blood; LN, Lymph node; PE, pleural effusion
